# An Unusual Presentation of Giant Cell Tumour of Bone in the Talus: A Case Report and Literature Review

**DOI:** 10.7759/cureus.42138

**Published:** 2023-07-19

**Authors:** Brandon Lim, Andrea Mc Carthy, Johnny Mc Kenna

**Affiliations:** 1 School of Medicine, Trinity College Dublin, Dublin, IRL; 2 Department of Trauma and Orthopaedic Surgery, St James’s Hospital, Dublin, IRL

**Keywords:** gct, tarsal bones, bone tumour, talus, giant cell tumour

## Abstract

Giant cell tumours (GCTs) of the bone often arise in the long bones while occurrence in smaller bones of the hand and feet is very rare. We report a case of GCT in the talus of a 17-year-old male who presented with a six-month history of worsening pain in his left ankle and loss of function, reducing his ability to walk and participate in sports. Radiographs of the ankle showed bony overgrowth on the head and neck of the talus with cortical breaching. MRI revealed possible extension into soft tissue and bone marrow oedema. CT scan also revealed an aggressive lytic lesion at the head and neck of the talus. He was managed with intralesional curettage and autologous bone grafting with bone harvested from the left knee. There was no evidence of recurrence at the six-month follow-up and the patient was able to walk freely. In conclusion, GCTs of the talus tend to occur in younger and healthier patients and have disastrous consequences if they persist, recur, or metastasize. Given the severe negative impact that GCTs have on a patient’s quality of life, they must be ruled out when investigating any ankle pain or reduced mobility. Current treatment options have produced consistently positive results while novel therapies that enable a faster return to weight bearing and reduce recurrence appear promising.

## Introduction

Giant cell tumours (GCTs) of the bone are benign but potentially aggressive tumours more commonly seen in women and arise between the ages of 20 and 40 years [1]. GCTs most commonly occur in the epiphysis of long bones such as the distal femur, proximal tibia, distal radius, and sacrum while GCTs of the tarsal and metatarsal bones are rare, occurring in only 1.5% of cases [2,3]. GCTs present with pain, swelling, and reduced range of motion [1]. GCTs are radiolucent on radiography and can be differentiated from other benign bone lesions using their incomplete sclerotic rim, while on histology, they display numerous giant cells with benign spindle cells [1]. The traditional management of GCTs using intralesional curettage and bone grafting has a 27-60% local recurrence rate in a period ranging from two to 30 years [4]. There is a risk of lung metastases, seen in 1-6% of cases and usually between 18 and 24 months, hence requiring routine chest radiographs [1,5]. We present an unusual presentation of a GCT of the bone in the talus of an adolescent male and review the literature on GCTs presenting in the talus.

## Case presentation

A 17-year-old male presented with a six-month history of worsening pain in his left ankle and foot with increasing loss of function, becoming most severe two months prior. There was an associated reduction in left lower limb muscle bulk. The patient who was fit and active was now struggling to walk and was unable to play sports for the past six months. He complained of poor sleep and a weight loss of 5 kg. His appetite was normal, and he denied any history of trauma, fever, night-time pain, or night sweats. There was also no right ankle pain and no left hip or knee pain. On examination, there was diffuse dorsal-medial-lateral swelling of the left ankle. He had a full range of motion, intact distal neurovascular status and no overlying skin changes, redness or warmth. He was walking with an antalgic gait and was unable to run.

**Figure 1:**
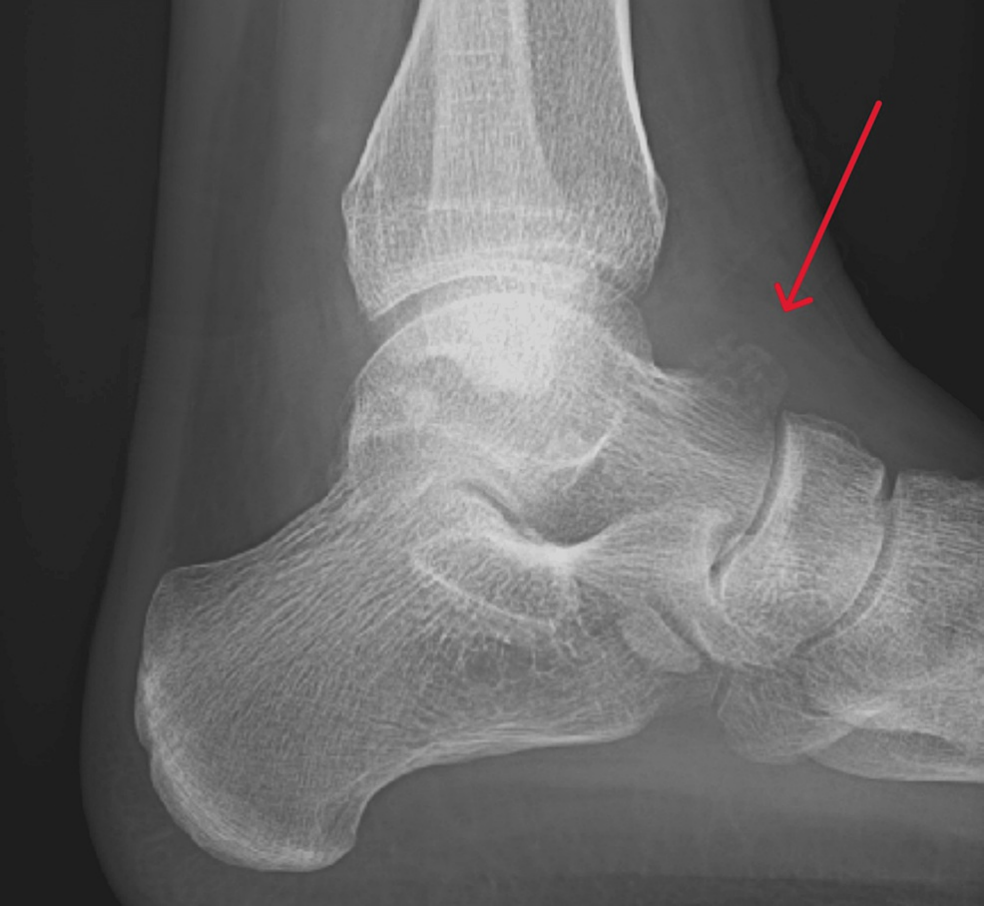
Radiograph of the left ankle showing lucency overlying the anterior talus with surrounding bony formation

**Figure 2:**
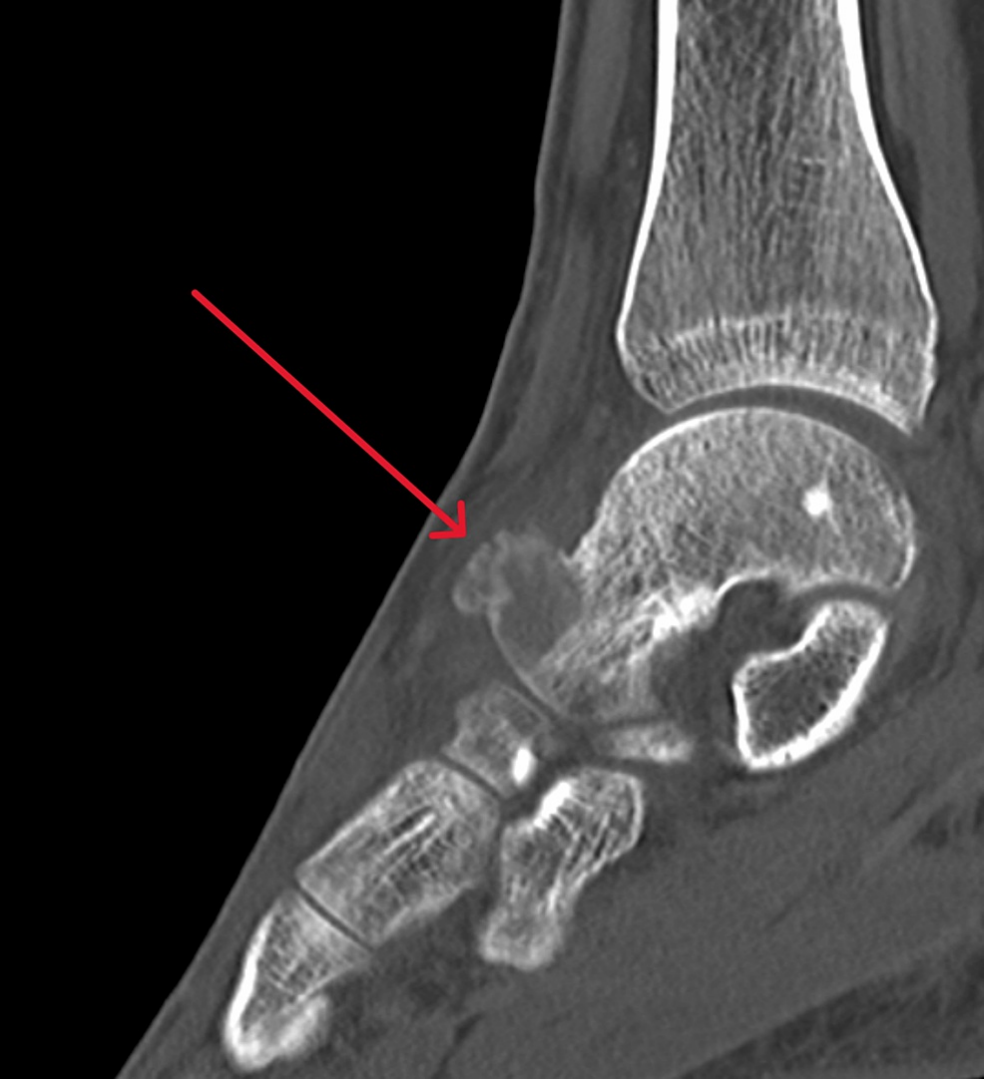
CT scan of the left ankle showing a solitary hypoattenuating bone lesion at the supralateral head and neck of the talus with adjacent periosteal reaction and lucency extending to the cortex

**Figure 3:**
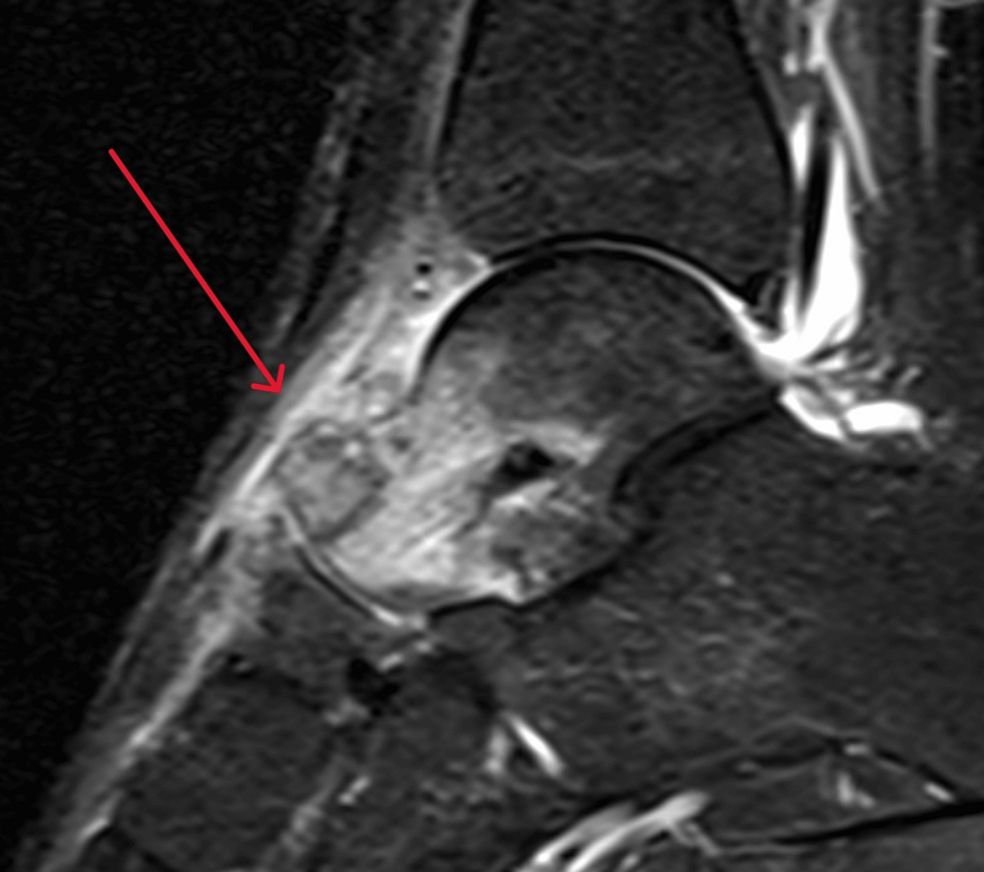
MRI of the left ankle showing the lesion, soft tissue reaction and bony oedema

Radiographs of the left ankle showed bony overgrowth on the head and neck region of the talus with cortical breaching (Figure 1). A computed tomography (CT) scan revealed an aggressive 20 mm lytic lesion at the head and neck of the left talus (Figure 2). Magnetic resonance imaging (MRI) revealed possible extension into soft tissue, a subcortical bone structure of about 1.5 x 1 x 1.2 cm in size, a small defect in the cortical layer and extensive bony oedema (Figure 3). Apart from neutrophilia at 7.7×10⁹/L, general haematology results, such as haemoglobin, white cell count and erythrocyte sedimentation rate, were normal. Biochemistry results revealed high alkaline phosphatase at 161 IU/L and high calcium at 2.6 mmol/L. C-reactive protein and lactate dehydrogenase were normal. A coagulation screen revealed a prothrombin time of 14.2 s.

The expertise and guidance of the tertiary-level orthopaedic oncology centre were sought, and the decision was made to proceed with an ultrasound-guided biopsy to rule out osteosarcoma. An orthopaedic consultant specialist in foot and ankle surgery performed the procedure in consultation with the orthopaedic oncology surgical specialist. This was done under aseptic conditions using a 13 gauge x 16 cm bone biopsy needle to access the lytic lesion of the anterior medial talus. Multiple samples were taken directly to histology. Results indicated that the samples received were non-representative of the lesion in question. A supplementary report further stated that there were some fragments of slightly reactive bone and normal articular cartilage. Although the biopsy did not identify any inflammation or tumour, the radiologists reported there was a significant lesion at the time of biopsy, suspected to be a GCT or chondral blastoma. These results thus fell into the 10% of oncological biopsies that are deemed non-conclusive. Based on this finding, the tertiary centre was contacted again and the decision to operate and remove the lesion was made.

**Figure 4:**
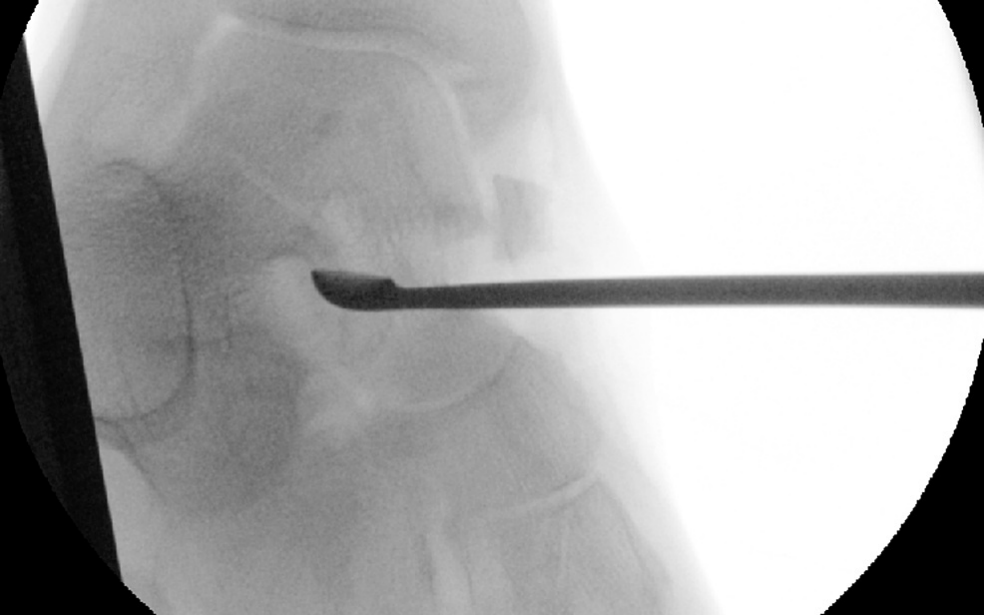
Intra-operative image of the lesion being curetted out

A left ankle excisional bone biopsy of the talus with bone grafting and left knee bone graft harvesting was conducted. Under the sterile technique, a tourniquet was inflated to 300 mmHg and the patient was placed in the supine position with standard draping. The talar neck was approached by a standard dorsomedial approach. Samples were curetted and sent for histopathology tests and tissue culturing (Figure 4). The anteromedial knee was approached via an oblique incision and bone was harvested from the medial proximal tibia. This was later grafted into the talar defect. The procedure was completed by closing the talar window, washout and subcutaneous and skin closure. Post-operatively, compression stockings were used followed by compression banding and a fracture boot, allowing for full weight bearing. The patient had two doses of antibiotics and started mechanical and chemical anticoagulants and pain management. He was instructed to return for re-dressing in one week and for a review and stitch removal in two weeks. The patient was non-weight bearing for two weeks post-surgery until he was seen at the outpatient department, after which he was allowed to begin progressive weight-bearing.

Postoperative histopathology reported a 2 cm benign bone-forming tumour characterised by numerous bony trabeculae with variable osteocyte rimming and prominent vascular stroma. The tumour was shown to have mixed histology containing a significant amount of osteoclast-type giant tumour cells with no background stromal cells. These were consistent with type III cells found in benign GCTs. Results of soft tissue culturing showed no evidence of infiltration on return.

**Figure 5:**
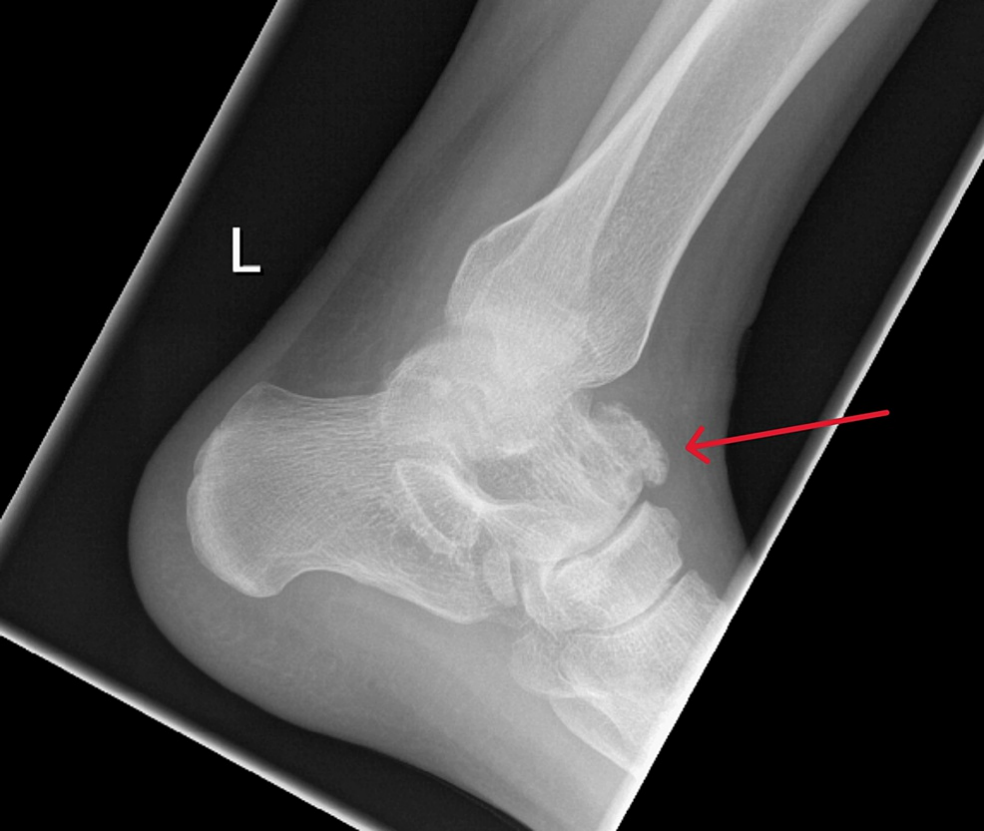
Six-month post-operative radiograph showing the bone graft

The patient was seen at the outpatient department six weeks post-operatively. The surgical wound was healing well, and he was able to walk freely with no complaints. At the six-month follow-up, the patient was well and there was no radiographical evidence of recurrence or metastasis (Figure 5). A plan was made for him to continue with physiotherapy and return to the outpatient department in one year.

## Discussion

A literature search was conducted on PubMed using the following combination of keywords: “giant cell tumour”; or “giant cell tumor”; or “GCT”, and “talus”. Results were restricted to English publications with available full-texts and case reports involving GCTs of the talus without any date restrictions. A summary of 16 cases reported in 15 studies can be found in Table 1.

**Table 1:** Summary of the literature ROM = range of motion

Author (Year)	Age (Yrs)	Gender	Duration of Symptoms	Location	Management	Follow-up & Outcome
Bapat et al., (2000) [6]	17	F	2 months.	Right talus (body).	Intralesional curettage, autologous bone grafting.	6 months: no evidence of recurrence, normal ankle ROM, minimal restriction of subtalar ROM.
Chaudhary et al., (2022) [7]	17	F	10 months.	Left talus (whole talus).	Talectomy and tibiocalcaneal fusion.	9 years: no evidence of recurrence.
Galvan et al., (2020) [5]	43	F	1 month.	Left talus (neck).	Intralesional curettage, adjuvants (high-speed burr, dilute hydrogen peroxide, sterile water, argon beam), bone cement filling.	2 years: no evidence of recurrence or bone resorption around the cement.
Kadam & Dhamangaonkar (2016) [8]	21	M	6 months.	Right talus (body; posteromedial aspect).	Intralesional curettage, chemical cauterization with phenol, autologous bone grafting (ipsilateral iliac crest).	2 years: no evidence of recurrence, good consolidation of the bone graft, no signs of collapse of the weight-bearing articular surface.
Kumar et al., (2014) [9]	62	M	1 month.	Right talus.	Talectomy and tibiocalcaneal fusion.	6 months: increased pain and swelling, recurrence and metastasis were confirmed, required a below-knee amputation.
						2 years: no recurrence, chest metastasis did not progress.
Malawer & Vance (1981) [10]	22	M	2 years (initially attributed to a sprain).	Right talus (head and neck).	Intralesional curettage, triple freeze-thaw cycle of liquid nitrogen, autologous bone grafting (contralateral iliac crest).	2 years: free of local disease.
	25	M	1 year.	Left talus (head and neck).	En bloc resection of the talar neck with the fusion of the talonavicular joint, autologous bone grafting (iliac crest).	5 years: asymptomatic, ankle ROM lacked 15° of full dorsiflexion and 10° of inversion and eversion. No radiographical evidence of local recurrence.
Mechlin et al., (1984) [11]	18	F	6 months.	Left talus.	En bloc resection, tibiocalcaneal fusion, autologous bone graft (iliac bone).	9 months: no evidence of recurrence.
Mella et al., (1982) [12]	13	F	1 year.	Right talus (anterior aspect).	Intralesional curettage, packing with autologous bone (right iliac crest) and homologous bone from a bone bank.	9 months: recurrence, below-knee amputation.
						21 months: radiographical evidence of lung metastasis, chemotherapy commenced.
						27 months: thoracotomy, no recurrence 12 months afterwards.
Panda et al., (2022) [13]	22	F	6 months	Left talus (body; anterolateral aspect).	Intralesional curettage, bone cement filling.	2 years: no evidence of recurrence or metastasis.
Sakamoto et al., (2020) [14]	25	F	6 months.	Left talus (lateral side).	Intralesional curettage, reconstruction with low porosity β-TCP blocks.	2 years: no evidence of recurrence, normal gait.
Schoenfeld et al., (2007) [15]	34	F	Diagnosed 1 year earlier, 2 failed attempts at curettage and bone grafting.	Right talus (medial aspect).	Intralesional curettage, reconstruction with a fresh-frozen osteochondral allograft.	12 months: pain-free, able to fully participate in all activities of daily living.
Selek et al., (2007) [16]	31	M	3 years.	Left talus (head and neck).	Intralesional curettage, electrocauterization and chemical cauterization with hydrogen peroxide, filling with cancellous bone allograft and demineralized bone matrix.	25 months: asymptomatic, no pain or restricted ROM. Radiographs showed a well-incorporated and remodelled graft, no evidence of recurrence.
Song & Park (2015) [4]	30	F	Several years.	Right talus (body).	Intralesional curettage, autologous bone grafting (iliac bone).	19 years: no evidence of relapse or changes in symptoms.
Varshney et al., (2010) [17]	14	M	3 months.	Left talus and calcaneum.	Intralesional curettage, coralline-based hydroxyapatite synthetic bone graft.	2 years: no radiographical evidence of recurrence or metastasis, symptom-free, nearly full ROM in subtalar and ankle joints.
Yang et al., (2021) [18]	22	M	6 months.	Left talus.	Talectomy, reconstruction using a 3D-printed talar prosthesis.	6-months: able to move ankle, mild pain during sports.
						12 months: nearly full ROM and grasp force of the ankle.

Among 16 case studies, 56% of patients were within the 20-40-year-old age range [4,8,10,13-16,18] while 31% were under 20 [6,7,11,12,17] and 13% were over 40 [5,9]. The ratio of male to female cases was 7:9 and the duration of symptoms ranged from one month to three years. Since the patient in our case falls in the under-20 category and there are several similar cases in this age group, it should be noted that younger patients seem to be more prone to GCTs of the small bones of the hand and foot, with Chaudhary et al., (2022) stating that 30% of GCTs of bones of the foot are seen in those under-20 [6,7]. GCTs of the foot also tend to be more aggressive than GCTs of long bones with a higher chance of multicentricity [6,7,17]. Varshney et al. (2010) discuss a case of multicentric GCT in the immature skeleton, highlighting that epiphysial plate involvement is associated with the tumour’s aggressiveness, and multicentric lesions have a higher risk of metaphyseal involvement and pathological fractures [17]. Given how GCTs of the tarsal bones affect younger and healthier patients, an aggressive tumour localised to an articular region can greatly reduce a patient’s quality of life, increasing their risk of fractures, irreparable osseous destruction and metastasis to other organs [12].

Like the patient in this case, the head and neck of the talus were specifically affected in five cases [5,7,10,16] while Varshney et al. (2010) also mention calcaneal involvement [17]. According to Schoenfeld et al. (2007), GCTs affect the calcaneus and metatarsals more than the talus but when the talus is involved, the tumour is most likely localised to the head and neck [15]. Mechlin et al. (1984) stated that among 28 cases of GCT in the tarsal bones, 13 were in the talus, 11 were in the calcaneus, two were in the navicular, one was in the cuboid, and one was in the middle cuneiform [11].

Physical examination findings reported include pain, swelling and tenderness [5-10,12,13,15-18]; an antalgic gait [6,10]; and painful and/or restricted range of motion at the ankle joint [4,7,8,10,13,15,16,18] or subtalar joint [6,8,13,15,16,18]. These symptoms are often severe enough to interfere with daily activities such as walking or a patient’s ability to do sports. This was consistent with the patient presented in our case.

Our case utilised radiography, MRI and CT scans to identify the lesion and assess the extent of its growth and spread. In the literature, radiographs were similarly useful in 14 cases [4,6-16,18], were unremarkable in one case [17] and were not used in one case [5]. Radiography was also useful in assessing any narrowing of joint spaces [4,6,10,13]. Furthermore, in the case of Kumar et al. (2014), evidence of a cortical breach allowed for the case to be managed without using MRI or CT scans [9]. GCTs in the tarsal bones often appear as osteolytic lesions without periosteal new bone formation on radiography [11]. MRI was used in seven cases [4,5,8,13,14,16,18] and CT scans were used in eight cases [5,7,11,13-15,17,18]. In addition to visualising the lesion, MRI was also able to identify bone marrow oedema [5,8,16] and intralesional haemorrhage [13,14]. Biopsies were used to confirm GCT in nine studies [4,5,10-14,16,18]. Histology results revealed either mononucleated or multinucleated giant cells with spindle cells in the background [5,11,13,14,16]. Fine-needle aspiration cytology was also used in three studies to diagnose a GCT [7-9]. Other investigations include general body examinations and chest radiographs to rule out clinical signs of infection, multicentric disease, pulmonary metastasis, and dermal, ocular or skeletal anomalies [4,8,9,11,13,17]; and biochemistry studies, including haemoglobin, white cell count, erythrocyte sedimentation rate, C-reactive protein, blood glucose, serum acid or alkaline phosphatase levels, serum calcium levels, serum parathyroid hormone levels, serum phosphorus levels and renal and hepatic profiles [4,6-11,13,16-18].

Similar to how the patient in our case was managed using intralesional curettage and autologous bone grafts harvested from his knee, six cases used curettage and bone grafts taken from sites such as the iliac crest [4,6,8,10,12,16] while two used bone cement filling [5,13], one used a synthetic bone graft [17], one used low porosity β-TCP blocks [14], and one used a fresh-frozen osteochondral allograft [15]. Alternatively, three cases involved full talectomies followed by talocalcaneal fusion [7,9] or reconstruction with a 3D-printed prosthesis [18], and two cases involved en bloc resections followed by fusion of the talonavicular joint [10] or tibiocalcaneal fusion [11] with subsequent autologous bone graft. Should surgery be unfeasible, the next course of action involving radiotherapy and denosumab treatment carries the risk of malignant transformation due to radiation [13]. According to Panda et al. (2022), the benefits of bone cement filling overcome the shortfalls of bone grafts, enabling immediate structural support for weight bearing, while producing a thermotoxic effect to kill residual tumours and allowing the early detection of any recurrence [13]. Adverse effects of bone cement are thermal necrosis and long-term degenerative changes in adjacent joints [13]. Novel management with a 3D-printed prosthetic reduces GCT recurrence and restores walking function [18].

Follow-up assessments include checking for physical signs or radiographical evidence of recurrence or metastasis, testing for improvement in ankle or subtalar joint range of movement, and assessing the patient’s ability to walk or carry out daily activity [4-18]. Routine follow-up is important because there is a 20% chance of local recurrence and a 5% chance of lung metastasis [13]. Positive outcomes were reported in 14 cases with post-operative follow-ups ranging from six months to nine years [4-8,10,11,13-18]. Kumar et al., (2014) reported a case where recurrence and pulmonary metastasis were detected at the six-month follow-up, requiring the need for a below-knee amputation to stop the progression of metastasis [9]. Mella et al. (1982) reported a case where recurrence detected at the nine-month follow-up warranted a below-knee amputation while lung metastasis detected at 21 months required chemotherapy and later, a thoracotomy [12]. Mella et al. (1982) further highlight the risk of mortality should the GCT undergo a malignant change or metastasize, quoting previous studies where 10 out of 18 patients died post-GCT diagnosis, and 18 out of 218 patients died after lung metastasis occurred [12].

## Conclusions

Given the higher risk of GCT in the tarsal bones in patients under 20 and the disastrous effect it can have on their quality of life, it must be detected early and removed before it becomes malignant or metastasizes. Traditional treatments of GCTs of the talus continue to appear effective while non-traditional management and novel therapies have also been reported to produce encouraging outcomes. The treatments may also allow recurrence to be detected earlier, reduce recurrence and enable earlier weight bearing and return to normal walking function.
